# Management of Chemotherapy Side Effects in Childhood Leukemia: Persian Medicine Experience

**DOI:** 10.31661/gmj.v12i.2926

**Published:** 2023-06-10

**Authors:** Babak Daneshfard, Amir Mohammad Jaladat, Majid Nimrouzi

**Affiliations:** ^1^ Chronic Respiratory Diseases Research Center, National Research Institute of Tuberculosis and Lung Diseases (NRITLD), Shahid Beheshti University of Medical Sciences, Tehran, Iran; ^2^ Persian Medicine Network (PMN), Universal Scientific Education and Research Network (USERN), Tehran, Iran; ^3^ Research Center for Traditional Medicine and History of Medicine, Department of Persian Medicine, School of Medicine, Shiraz University of Medical Sciences, Shiraz, Iran

**Keywords:** Chemotherapy, Leukemia, Integrative oncology, Pediatrics, Persian Medicine

## Dear editor,

Leukemia is the most common (up to 40%) cancer in children among which, acute lymphoblastic leukemia accounts for about 80% of them. Although the annual incidence rate of leukemia is 39 per million children, it has had an increasing trend during the last decades [1][2].

Various side effects have been reported as complications of cancer treatment in children including gastrointestinal disorders, oral lesions, peripheral neuropathy, pulmonary infections, neutropenia, hepatotoxicity, cardiotoxicity, endocrine disturbances, and thromboembolic complications. These side effects not only decrease the quality of life but also negatively affects the response rate of the treatment [3].

Nowadays, various complementary and alternative medicines are integratively used in an evidence-based manner in order to shorten the disease course and decrease treatment-induced complications [4]. Although few studies have investigated the efficacy of complementary methods in children with cancer, integrative pediatric oncology is an emerging field that explores the use of complementary and alternative medicine (CAM) in pediatric oncology [5].

Persian medicine (PM) is one of the oldest comprehensive schools of medicine with thousands-of-years history [6]. In addition to its preventive health measures which are considered as its important basics, PM recommends simple useful therapeutic recommendations for various medical conditions including cancer [7].

Many of these treatments for cancer patients have been investigated through clinical trials with promising results [8][9]. For instance, we have examined the PM therapeutic approach for the management of chemotherapy induced neutropenia and liver toxicity in pediatric leukemic patients.

Chemotherapy induced neutropenia (CIN) is one of the most common and most serious complications of cancer treatment. This hematologic toxicity predisposes the patients to other complications including various infectious diseases. In a randomized placebo-controlled trial, we investigated the efficacy of a natural syrup from chamomile to control this side effect in pediatric leukemic patients [10]. Comparison of the absolute neutrophil count (ANC) between the groups revealed a significant improvement and faster recovery from neutropenia in the treatment group during and after one month of concomitant complementary treatment.

Another frequent complication of chemotherapy in children is liver toxicity. In addition to having no effective preventive measure, this side effect could be a hindrance of a successful chemotherapy. In line with our clinical experience in pediatric oncology, we reported a successful treatment of a 5-year-old girl with acute lymphocytic leukemia who had been presented with chemotherapy-induced liver injury. PM treatment period included 15 days consumption of Nogho-e-Favakeh or soaked fruits (a juice consisted of prune, tamarind, brown sugar, and manna of Hedysarum) followed by one-month oxymel and chicory distilled water complementary to her chemotherapy regimen. Improvement of liver enzymes (SGOT and SGPT) continued all through the 3-month follow-up reaching to the normal range [11].

Fever is also a common complication in children with cancer as a result of either their disease or the received chemotherapy drugs. An interesting trial on pediatric cancer patients with febrile neutropenia revealed that using a topical oil from Viola odorata (rubbing 20 drops of oil on periumbilical area) not only could dramatically decrease the temperature only after 30 minutes, but also decrease the need for rescue treatment with antipyretic medication [12]. In fact, this finding is promising for further investigations on the efficacy of topical dosage forms in cancer patients.

All in all, it seems that there is a great potential in PM for clinical management of cancer patients in order to (at least) decrease their complications and consequently, increase their quality of life. Future investigations are guaranteed to evaluate the safety and efficacy of these recommendations.

## Conflict of Interest

None.

## References

[R1] Karalexi MA, Tagkas CF, Markozannes G, Tseretopoulou X, Hernández AF, Schüz J, et al (2021). Exposure to pesticides and childhood leukemia risk: A systematic
review and meta-analysis. Environ Pollut.

[R2] Marcotte EL, Spector LG, Mendes-de-Almeida DP, Nelson HH (2021). The prenatal origin of childhood leukemia: Potential applications
for epidemiology and newborn screening. Front Pediatr.

[R3] Ruggiero A, Skinner R, Khaled Zekri (2021). Adverse and Toxic Effects of Childhood Cancer Treatments. Front Oncol.

[R4] Daneshfard B, Sanaye MR, Nimrouzi M (2019). Prolegomena to a true integrative medical paradigm. Altern Ther Health Med.

[R5] Längler A, Mansky PJ, Seifert G (2013). Integrative Pediatric Oncology. Springer Berlin Heidelberg.

[R6] Daneshfard B, Sadr M, Abdolahinia A, Azari H, Naseri M, Iranzadasl M, et al (2022). Mansur ibn Ilyas Shirazi (1380–1422 AD), a pioneer of
neuroanatomy. Neurol Sci.

[R7] Mahjour M, Khoushabi A, Noras MR (2018). Herbal Remedies for Cancer based on Persian Medicine. TIM.

[R8] Shahriari M, Azadbakht M, Roohparvar M, Daneshfard B, Majid N (2021). Effect of chamomile on chemotherapy-induced neutropenia: a pilot
open-label controlled trial. PJMHS.

[R9] Soltani GM, Hemati S, Sarvizadeh M, Kamalinejad M, Tafazoli V, Latifi SA (2020). Efficacy of the plantago major L syrup on radiation induced oral
mucositis in head and neck cancer patients A randomized, double blind,
placebo-controlled clinical trial. Complement Ther Med.

[R10] Daneshfard B, Shahriari M, Heiran A, Nimrouzi M, Yarmohammadi H (2020). Effect of chamomile on chemotherapy-induced neutropenia in
pediatric leukemia patients: A randomized triple-blind placebo-controlled
clinical trial. Avicenna J Phytomed.

[R11] Jaladat A-M, Zareifar S, Daneshfard B, Shahriarirad R (2022). Management of Drug-Induced Liver Injury with Persian Medicine:
Case Report. Integr Med Rep.

[R12] Tafazoli V, Shahriari M, Heydari M, Nikbakht HA, Zarshenaas MM, Nimrouzi M (2020). The Effect of Viola Odorata L Oil for Fever in Children A
Randomized Triple-blinded Placebo-controlled Clinical Trial. Curr Drug Discov Technol.

